# Auxiliary Subunits Regulate the Dendritic Turnover of AMPA Receptors in Mouse Hippocampal Neurons

**DOI:** 10.3389/fnmol.2021.728498

**Published:** 2021-08-23

**Authors:** Ali Harb, Nils Vogel, Ali Shaib, Ute Becherer, Dieter Bruns, Ralf Mohrmann

**Affiliations:** ^1^Zentrum für Human- und Molekularbiologie, Saarland University, Homburg, Germany; ^2^Department of Anaesthesiology, University Medical Center Göttingen, Göttingen, Germany; ^3^Institute for Physiology, Otto-von-Guericke University, Magdeburg, Germany; ^4^Institute of Neurophysiology, University Medical Center Göttingen, Göttingen, Germany; ^5^Institute for Physiology, Center for Integrative Physiology and Molecular Medicine, Saarland University, Homburg, Germany; ^6^Center for Behavioral Brain Science, Otto-von-Guericke University, Magdeburg, Germany

**Keywords:** AMPA receptors, TARPgamma-8, CKAMP44, recycling endosomes, receptor turnover

## Abstract

Different families of auxiliary subunits regulate the function and trafficking of native α*-amino-3-hydroxy-5-methyl-4-isoxazolepropionic acid* (AMPA) receptors in the central nervous system. While a facilitatory role of auxiliary subunits in ER export and forward trafficking of newly synthesized AMPA receptors is firmly established, it is unclear whether auxiliary subunits also control endosomal receptor turnover in dendrites. Here, we manipulated the composition of AMPA receptor complexes in cultured hippocampal neurons by overexpression of two auxiliary subunits, *transmembrane AMPAR regulatory protein* (TARP) γ-8 or *cysteine knot AMPAR-modulating protein* (CKAMP) 44a, and monitored dendritic receptor cycling in live-cell imaging experiments. Receptor surface delivery was assayed using a modified AMPA receptor subunit carrying the pH-dependent fluorophore *superecliptic pHluorin* (SEP-GluA1), which regains its fluorescence during receptor exocytosis, when transiting from the acidic lumen of transport organelles to the neutral extracellular medium. Strikingly, we observed a dramatic reduction in the spontaneous fusion rate of AMPA receptor-containing organelles in neurons overexpressing either type of auxiliary subunit. An analysis of intracellular receptor distribution also revealed a decreased receptor pool in dendritic recycling endosomes, suggesting that incorporation of TARPγ-8 or CKAMP44a in receptor complexes generally diminishes cycling through the endosomal compartment. To directly analyze dendritic receptor turnover, we also generated a new reporter by N-terminal fusion of a self-labeling HaloTag to an AMPA receptor subunit (HaloTag-GluA1), which allows for selective, irreversible staining of surface receptors. Pulse chase-experiments with HaloTag-GluA1 indeed demonstrated that overexpression of TARPγ-8 or CKAMP44a reduces the constitutive internalization rate of surface receptors at extrasynaptic but not synaptic sites. Thus, our data point to a yet unrecognized regulatory function of TARPγ-8 and CKAMP44a, by which these structurally unrelated auxiliary subunits delay local recycling and increase surface lifetime of extrasynaptic AMPA receptors.

## Introduction

α*-Amino-3-hydroxy-5-methylisoxazole-4-propionic acid* receptors (AMPARs) constitute the primary class of ligand-gated cation channel found at central glutamatergic synapses. Dynamic recruitment of AMPARs to synaptic sites putatively underlies fundamental forms of synaptic plasticity (Herring and Nicoll, 2016; Park, 2018). The channel-forming core of AMPARs consists of four multi-domain subunits, each containing three transmembrane domains and a membrane-inserted loop that lines the inner channel pore (Dingledine et al., 1999; Traynelis et al., 2010). Native AMPARs generally contain two GluA2 subunits, which render receptors Ca^2+^-impermeable and determine single channel conductance and rectification (Cull-Candy et al., 2006; Isaac et al., 2007). Most neurons express only marginal amounts of GluA2-lacking AMPARs with Ca^2+^-permeability that have been proposed to play a role in the early phases of NMDA receptor-dependent long-term potentiation, when they are delivered to the surface and transiently populate synaptic sites (Swanson et al., 1997; Cull-Candy et al., 2006).

The core channel complex is surrounded by auxiliary subunits that modulate its functional properties (Jackson and Nicoll, 2011; Straub and Tomita, 2012; Haering et al., 2014). *Transmembrane AMPAR regulatory proteins* (TARPs) currently constitute the best characterized family of auxiliary subunits. This group of subunits comprises two classes of related tetraspan membrane proteins (type I: TARPγ-2/-3/-4/-8 and type II: TARPγ-5/-7) with homology to claudins and calcium channel γ-subunits. The prototypical TARP type I isoform stargazin (TARPγ-2), which is ubiquitously expressed in neurons, has been demonstrated to alter channel properties in favor of the open pore state by slowing deactivation and decreasing desensitization (Priel et al., 2005; Tomita et al., 2005; Turetsky et al., 2005). As a secondary function, stargazin is also required for normal surface expression of AMPARs, which is particularly evident in cerebellar granule cells of stargazer mice, wherein surface expression and synaptic targeting of AMPARs are dramatically reduced due to dysfunctional stargazin (Chen et al., 2000). Mechanistically, TARP association was proposed to support AMPAR trafficking by masking ER retention signals (Vandenberghe et al., 2005a,b; Bedoukian et al., 2006, 2008) and providing additional export/sorting motifs (Bedoukian et al., 2008). Increased forward trafficking could also indirectly result from the positive modulation of channel properties, which might increase the probability to pass functionality checkpoints during receptor export (Penn et al., 2008). Stargazin like other type I TARPs possesses a C-terminal PDZ ligand, which can interact with PDZ-domain-containing proteins. In particular, interactions of stargazin with the PDZ domains of the synaptic scaffolding protein PSD95, which belongs to a family of membrane-associated guanylate kinases and is almost exclusively localized in the postsynaptic density of glutamatergic synapses (Won et al., 2017), were reported to be essential for the normal anchorage of AMPARs at postsynaptic sites (Chen et al., 2000; Schnell et al., 2002).

While not nearly as well characterized as stargazin, TARPγ-8 and other group I TARPs (γ-3/4) seem to serve similar but not identical functions (Tomita et al., 2003; Cho et al., 2007). TARPγ-8 like stargazin interacts with tetrameric AMPARs in variable stoichiometry in different neuron types, most likely two or four copies per receptor complex (Shi et al., 2009). Genetic ablation of TARPγ-8, which is preferentially expressed in the hippocampus, largely diminishes the extrasynaptic AMPAR pool and moderately decreases synaptic receptor accumulation (Rouach et al., 2005). Moreover, TARPγ-8 supports the expression of synaptic plasticity (Rouach et al., 2005), with CaMKII-mediated phosphorylation of TARPγ-8 constituting a major mechanistic step in the induction of receptor recruitment to synaptic sites (Park et al., 2016). Interestingly, phosphorylation-induced interactions between TARPγ-8 and PSD95 are important for basal synaptic transmission but are not strictly required for the expression of LTP (Sumioka et al., 2010, 2011).

TARP-mediated effects on AMPAR properties seem to be in part mimicked and in part antagonized by other auxiliary subunits belonging to structurally diverse protein families like *cysteine knot AMPAR-modulating proteins* (CKAMPs), cornichons, and *Germ cell-specific gene 1-like protein* (GSG1L) (Haering et al., 2014). Several members of the CKAMP/SHISA family have recently been proposed to play critical roles in regulating AMPAR function (reviewed in von Engelhardt, 2019), although they have been found at lower abundance than TARPs (Schwenk et al., 2014). CKAMPs generally constitute single-pass transmembrane proteins carrying a cysteine-rich extracellular domain and a large intracellular domain with a C-terminal PDZ-type II interacting motif. The four CKAMP family members (CKAMP39/44/52/59) are differentially expressed across brain regions, with CKAMP44 and CKAMP52 generally exhibiting the highest expression levels (Chen et al., 2014; Schwenk et al., 2014). Two splice variants have been identified for CKAMP44, CKAMP52, and CKAMP59, but the functional implications of the short cytosolic motifs that are encoded by the alternatively spliced exon have not been investigated so far (von Engelhardt et al., 2010; Farrow et al., 2015). Like other auxiliary AMPAR subunits, CKAMPs characteristically modulate gating properties of AMPARs: CKAMP44 decreases receptor deactivation rate, elevates desensitization rate, and delays recovery from desensitization (von Engelhardt et al., 2010; Khodosevich et al., 2014; Chen et al., 2018). Increased synaptic short-term facilitation in CKAMP44^–/–^ mice was largely attributed to the reduced desensitization of receptors lacking this auxiliary subunit. In contrast, short-term facilitation is either normal or decreased in synapses of CKAMP59^–/–^ (Schmitz et al., 2017) or CKAMP52^–/–^ mice (Klaassen et al., 2016), which points to potential isoform- and synapse-specific differences in the modulation of AMPARs. Although CKAMP44 has been shown to be of little consequence for the expression of LTP in the granule cells of the dentate gyrus (Khodosevich et al., 2014), recent work indicates that CKAMP52 and CKAMP59 are required for synaptic plasticity in cerebellar Purkinje cells (Peter et al., 2020) and hippocampal CA1 neurons (Schmitz et al., 2017), respectively. Altering the expression of CKAMP44 in neurons results in corresponding changes in AMPAR surface density (Khodosevich et al., 2014), which suggests that CKAMPs control AMPAR forward trafficking like TARPs. That said, different CKAMP isoforms have been shown to differentially support AMPAR surface expression, which possibly also reflects additional influences of AMPAR subunit composition and cell type on trafficking (von Engelhardt et al., 2010; Khodosevich et al., 2014; Chen et al., 2018).

A dynamic regulation of local AMPAR turnover is believed to underlie changes in postsynaptic receptor accumulation during synaptic plasticity (Henley and Wilkinson, 2013; Buonarati et al., 2019). It is well established that intracellular compartments, in particular dendritic recycling endosomes (REs), can rapidly supply AMPARs to the surface, from where the receptors are recruited to synaptic sites during LTP (Park et al., 2004, 2006). Even under basal conditions a continuous cycling of receptors between surface pool and intracellular compartments has been observed (Ehlers, 2000; Hirling, 2009), which suggests a dynamic equilibrium between receptor endocytosis and surface delivery. While auxiliary subunits have been implicated in ER export of AMPAR, it is unknown whether their presence in receptor complexes also affects local dendritic receptor turnover. Here, we have visualized internalization and delivery of AMPAR in murine hippocampal neurons in culture to investigate potential effects of TARPγ-8 or CKAMP44a on local receptor cycling. Intriguingly, we demonstrate that increasing the abundance of both auxiliary subunits delays the constitutive turnover of GluA1-containing AMPAR, as manifested in a reduced internalization rate of extrasynaptic receptors, a diminished membrane delivery frequency, and a decreased AMPAR population in REs. Thus, the association of AMPARs with specific auxiliary subunit types determines local turnover and receptor lifetime on the dendritic surfaces.

## Materials and Methods

### Expression Vectors

pCI SEP-GluA1 was a gift from Robert Malinow (Addgene plasmid # 24000; http://n2t.net/addgene:24000; RRID:Addgene_24000). Analogous to SEP-GluA1, we generated a new pCI HaloTag-GluA1-construct by insertion of the HaloTag^®^-sequence (Promega) after the signal peptide of GluA1 using PCR overlap extension. pCDNA3 TfR-tagRFPt was generated from pCDNA3 TfR-pHuji (kind gift by David Perrais; Addgene plasmid # 61505; http://n2t.net/addgene:61505; RRID:Addgene_61505) by in-frame replacement of pHuji with a PCR fragment encoding for tagRFPt. The expression vectors pRK5 TARPγ-8 and pRK5 CKAMP44a were kindly provided by Dr. J. von Engelhardt and were further modified in our lab by insertion of a PCR fragment encoding the fluorescent reporter NLS tdTomato under control of a Polio Virus IRES (inserted at *Sal*I restriction site). The cDNA for PSD95 was a kind gift by Dr. Oliver Schlüter and was C-terminally fused to tagRFPt by PCR overlap extension.

### Cell Culture and Transfection

Hippocampi were dissected from mice of either gender at P0–P4, dissociated, and kept in neuron-glia sandwich cultures as previously described (Kaech and Banker, 2006). All animals were handled in compliance with the federal German animal welfare act and local regulations at the University of Saarland and the Otto-von-Guericke University Magdeburg, respectively. To inhibit growth of astrocytes in the low density neuronal cultures on cover slips, the sandwich culture was treated with *5-Fluoro-2’deoxyuridine* (FUdR, Sigma, F0503) at 3 DIV. Neurons were transfected with expression vectors at 8–11 days (8–11 DIV) using calcium phosphate precipitation following standard protocols (Dudek et al., 2001). All imaging experiments were performed 24–48 h post transfection.

### Live-Cell Imaging of AMPAR Delivery

Epifluorescence imaging of SEP-GluA1-transfected hippocampal neurons was performed at an Olympus IX70 microscope with a 100 × 1.45 NA Plan Apochromat objective, a QuantEM 512SC camera (Photometrics), and a perfusion system. The EMCCD camera was also equipped with a Dual-View splitter (Visitron, Puchheim, Germany) for multicolor imaging (splitter cut-off at 590 ± 10 nm). Images were acquired at 10 Hz for 6 min using VisiView software (version 2.1.2; Visitron). All experiments were performed at room temperature in an extracellular solution (pH 7.4) containing (in mM): 145 NaCl, 2.4 KCl, 2 CaCl_2_, 1 MgCl_2_, 12 HEPES, and 10 D-Glucose. Image analysis was performed with *Fiji* software (ImageJ 1.52e) using custom macros. Delivery events were identified under visual inspection, and event kinetics was analyzed with a custom-written macro in Igor Pro 6 (Wavemetrics). To visualize intracellular REs containing SEP-GluA1, we transiently applied neutralizing NH_4_Cl-containing solution to dendrites and calculated difference images. Puncta-like SEP-GluA1 fluorescence signals in difference images were considered as intracellular SEP-GluA1-containing compartments, if NH_4_Cl-application led at least to a two-fold increase in fluorescence. Corresponding regions of interest (ROIs) were used on subtraction images (I_NH__4__Cl_ – I_baseline_) to determine the signal intensity of the SEP-GluA1-containing puncta. To estimate the density of REs per μm dendrite, REs were counted in continuous dendritic segments of at least 20 μm length.

### Live-Cell Imaging of AMPAR Internalization

To selectively label the surface pool of HaloTag-GluA1-containing AMPARs, transfected neurons were first incubated with the membrane-impermeable Alexa Fluor 488 ligand (Promega, G1001) for 35–40 min (in 5% CO_2_) at 15°C, in this way attenuating receptor endocytosis during the labeling interval. In order to distinguish the extrasynaptic receptor population from synaptically anchored receptors, neurons were co-transfected with the synaptic marker PSD95-tagRFPt. Confocal images of stained neurons were acquired with a laser scanning microscope (Zeiss LSM 780) using a temperature-controlled recording chamber. All images were acquired with a C-Apochromat 40 × 1.2 NA objective using a pinhole size of 1 AU, which corresponds to a slice thickness of 0.8 μm. During prolonged imaging sessions (24 min, image acquisition every 4 min) at 37°C we relied on the autofocus mechanism of the microscope to sufficiently maintain the focal plane. Images were acquired every 4 min. At each time point, three slices with 1 μm interval were acquired. The uptake of extrasynaptic receptors was quantified by measuring the fluorescence decay within dendritic membrane segments using a continuous line scan (length > 3 μm) in confocal slices. The region surrounding the line was transformed into a rectangular image (Kocsis et al., 1991), and all pixel lines of the transformed picture (fluorescence profiles) were averaged along the path. The averaged membrane peak fluorescence for each measured dendritic segment was used for further analysis. To quantify receptor turnover at synaptic sites, the PSD95-tagRFPt signals of all three confocal slices were summed and then thresholded to obtain ROIs corresponding to synaptic puncta. ROIs were overlaid on the summed image of HaloTag-GluA1 for every time point, and fluorescence intensity was quantified.

### Characterization of Receptor Surface Expression and Distribution

The subcellular distribution of SEP-GluA1-containing receptors was investigated using an epifluorescence Olympus BX51WI microscope with a 60 × 1.10 NA objective, CoolSNAP^2^ CCD camera (Photometrics), and a local perfusion system. To identify the surface AMPAR fraction, cells were superfused with extracellular solution of pH 5.5, and a difference image was generated by subtraction of the image under pH5.5 from a reference baseline image (I_surface_ = I_baseline_ − I_low pH_). Moreover, the total pool of AMPARs was visualized by application of NH_4_^+^-containing solution, which neutralizes intracellular compartments and renders all previously quenched SEP fluorescence visible. To estimate the fraction of surface AMPARs, we focused on the soma and calculated the ratio between the integrated fluorescence for the surface pool and total receptor pool. As receptors might not be evenly distributed on dendrites, we quantified the surface fluorescence along single dendrites for 70 μm starting at the soma. Dendrites were traced at their midline, and the surrounding image regions were transformed using the “straighten” command in ImageJ (Kocsis et al., 1991), such that the x-axis of the processed image was aligned with the main dendrite. Total fluorescence in each pixel row (pixel size: 0.215 μm) of the transformed image (perpendicular section through dendrite) was determined by a custom-written macro in Igor Pro 6 (Wavemetrics) in order to correlate the local membrane fluorescence with dendrite length. The integrated fluorescence intensity was normalized to the dendritic diameter at the corresponding position of the dendrite, as estimated by the distance between the extrema of the first derivative of the fluorescence signal (turning points). The longitudinal fluorescence profiles of all dendrites were fit by simple line functions, delivering slope and y-intercept as parameters.

### Statistical Analysis

Data are presented as mean ± SEM (standard error mean), unless stated otherwise in the text. For data with skewed distribution, the median or the average of medians across cells was used for statistical analysis. Statistical significance was tested in SigmaPlot software using Student’s two-tailed *t*-test between two groups, if normality could be assumed. To calculate statistical significance among three or more groups, we used one-way analysis of variance (ANOVA), if not stated otherwise. Significance level was assessed according to the following probability values: ^∗^*p* < 0.05; ^∗∗^*p* < 0.01; ^∗∗∗^*p* < 0.001.

## Results

### Constitutive Surface Delivery of AMPARs Is Mediated by Transient Fusion of Recycling Endosomes

**FIGURE 1:**
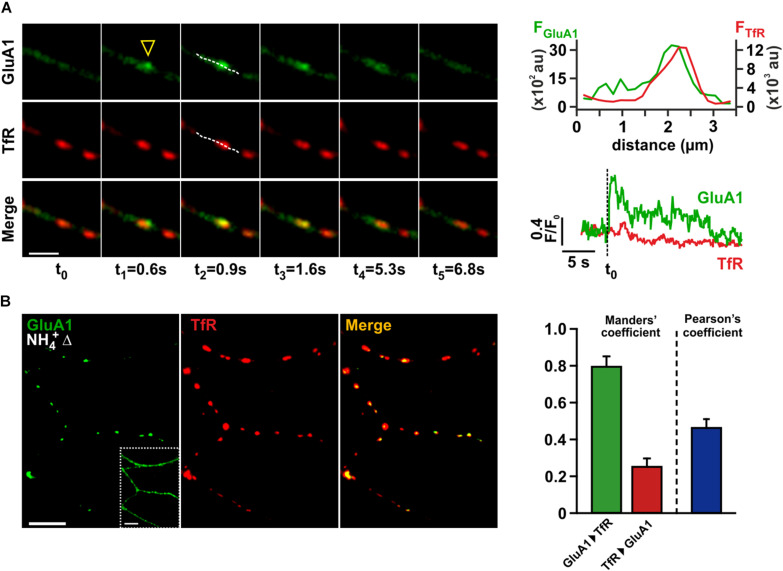
Kiss-and-run fusion of recycling endosomes underlies spontaneous AMPAR insertion in dendrites of hippocampal neurons. **(A)** Time series of consecutive images depicting an exemplary dendritic fusion event (scale bar = 4 μm). SEP-GluA1 fluorescence (green) and TfR-tagRFPt fluorescence (red) are shown in the upper and middle row, merged images in the bottom row. The fusion site is marked by a yellow arrowhead. Fusion events were classified as “RE-associated,” if line scans (hatched lines in images at t_2_) indicated a vast overlap of both signal peaks **(upper right panel)**. Temporal profiles for red and green signals in a region of interest enclosing the site of the fusion event are depicted in the lower right panel. **(B)** Intracellular AMPAR pools were analyzed by application of NH_4_^+^, allowing for visualization of intracellular AMPAR signals in a difference picture (NH_4_^+^Δ, left image; scale bars = 10 μm). The inset shows the same dendrites before NH_4_^+^-application, outlining the geometry of the dendritic arborization. Recycling endosomes within the same field of view were marked by TfR-tagRFPt (middle image). In order to quantify the signal overlap between tagged AMPARs and TfR-marked recycling endosomes, we calculated both Manders’ coefficents and Pearson’s coefficient (*n* = 11) **(right panel)**.

To study AMPAR trafficking in dendrites, we transfected low density hippocampal cultures with an expression vector encoding for a modified GluA1 subunit, which was N-terminally fused to the pH-dependent fluorophore *superecliptic pHluorin* (SEP-GluA1; Kopec et al., 2006). Live-cell imaging experiments were performed 24–48 h after transfection to allow for sufficient expression of SEP-GluA1. Due to the membrane topology of the GluA1-subunit, the SEP fluorophore at the N-terminal receptor domain is exposed to the acidic milieu in transport organelles, which almost completely quenches its fluorescence. Upon receptor exocytosis SEP fluorescence is restored, and the resulting local fluorescence transients can be used to detect surface receptor delivery (Yudowski et al., 2007; Lin et al., 2009). As we were especially interested in the dynamic re-insertion of AMPARs from REs in dendrites, we used a tagRFPt-tagged transferrin receptor variant (TfR-tagRFPt) to mark REs (Mukherjee et al., 1997) and recorded the spontaneous surface delivery of SEP-GluA1-containing AMPARs in dendritic segments within 2 min time intervals (Figure 1A). Local SEP fluorescence transients were classified as insertion events, if they exhibited a sudden rise in fluorescence (rise time < 2 s) and a peak fluorescence value >4-times baseline SD. In order to identify fusion events that originate from TfR-tagRFPt-positive REs, the movie frame showing the maximum SEP fluorescence of each event was superimposed onto the corresponding tagRFPt image. The positions of the event peak and nearby REs were compared by line scans running along the longitudinal axis of the dendrite (Figure 1A). tagRFPt-fluorescent puncta were only regarded as REs, if their peak fluorescence exceeded 4-times the SD of neighboring regions. Insertion events and REs were called “associated,” if the distance between both peak positions was less than the sum of the half-widths of the peaks, thereby demanding a substantial overlap of both signals. Using this definition, we found that 97% of all SEP-GluA1-delivery events (30 out of 31 events; 9 cells) were associated with TfR-tagRFPt-positive REs. Thus, the vast majority of fusion events originated from REs or substructures of REs.

The pH-independent RE-marker TfR-tagRFPt also allowed us to track the fate of a given RE after fusion, as a persistent TfR-tagRFPt signal after the decay of associated SEP-GluA1 fluorescence indicates kiss-and-run-type fusion. Interestingly, we never observed a total loss of tagRFPt fluorescence during fusion events that would suggest a complete collapse of the RE structure into the plasma membrane. We quantified the decay of TfR-tagRFPt peak fluorescence during each fusion event and also estimated fluorescence bleaching in a corresponding time window directly before the event. When calculating the bleaching-corrected relative change of tagRFPt fluorescence for each event, we found an average intensity decrease of 3.5 ± 1.2% (*n* = 30 events). Only 26.7% (8/30) of the associated fusion events exhibited a tagRFPt-fluorescence reduction that actually exceeded 5%. Although we cannot exclude that this subtle fluorescence loss is due to the full fusion of very small vesicular substructures belonging to the RE tubular network, we consider it more likely that RE integrity remains largely unchanged due to spontaneous fusion pore closure, in accord with earlier findings by Jullie et al. (2014). Therefore, the discharge of transmembrane cargo proteins during these fusion events is likely very limited, and the observed fluorescence decay of SEP-GluA1 may in part be due to a re-acidification of REs after re-closure of the fusion pore.

In view of the predominance of RE-associated fusion events in dendrites, we wondered about the fraction of intracellular SEP-GluA1-containing AMPARs that reside in REs under resting conditions. For visualization of the AMPARs in acidic organelles we applied NH_4_Cl (50 mM) solution to neurons co-expressing SEP-GluA1 and TfR-tagRFPt, as this treatment neutralizes the pH in all compartments and reconstitutes the fluorescence of contained tagged receptors. Difference images (denoted “NH_4_^+^Δ”) were generated to selectively show the fluorescence signal of this intracellular receptor population. Superposition of the difference images onto the corresponding TfR-tagRFPt-picture demonstrated a vast signal overlap (Figure 1B), suggesting that the majority of intracellular AMPARs is indeed found within dendritic REs. This observation is also reflected in a high mean Manders’ coefficient for the colocalization of SEP-GluA1 with TfR-tagRFPt (0.79 ± 0.05, n = 11). Since the mean Manders’ coefficient for the reverse comparison (TfR signal to GluA1 signal) was small (0.25 ± 0.04), and a relatively low Pearson’s coefficient was observed (0.46 ± 0.04), we conclude that SEP-GluA1-containing REs only constitute a subpopulation of all dendritic REs, in accord with previous results by Liu et al. (2016). Based on these data, the primary receptor pool that constitutively supplies AMPARs onto the surface maps to a rather limited subset of dendritic REs.

### Overexpression of TARPγ-8 or CKAMP44a Reduces the Frequency of Spontaneous Fusion Events

**FIGURE 2:**
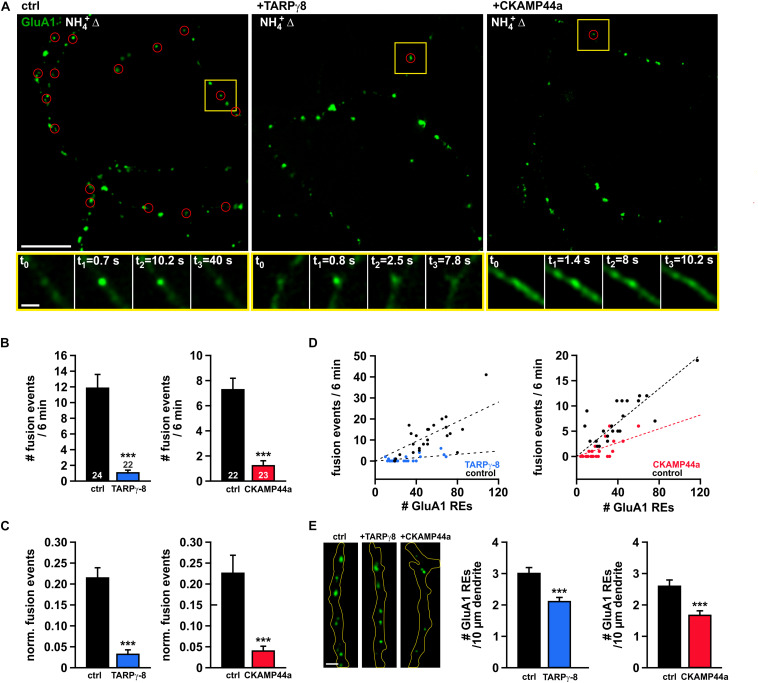
High abundance of TARPγ-8 or CKAMP44a diminishes spontaneous fusion events in dendrites. **(A)** Exemplary overlay images (scale bar = 10 μm) showing “visualized” intracellular SEP-GluA1-positive organelles (green) and corresponding sites of fusion events (red circles) that were identified during recording intervals of 6 min in controls **(left)**, neurons overexpressing TARPγ-8 **(middle)**, or neurons overexpressing CKAMP44a **(right)**. Yellow rectangles mark the position of exemplary fusion events that are shown in time series below each overview picture (acquisition frequency: 10 Hz). Scale bar of enlarged time series images is 2 μm. **(B)** Quantification of the average number of delivery events observed in 6 mins for dendritic structures from neurons overexpressing TARPγ-8 or CKAMP44a in comparison to controls. **(C)** Event frequencies were normalized to the number of identified intracellular AMPAR-containing organelles (recycling endosomes) in the field of view. **(D)** Plots of event frequency versus number of recycling endosomes for neurons overexpressing TARPγ-8, CKAMP44a, and corresponding controls. Note the altered relationship (slope of fit line) in neurons with increased auxiliary subunit levels. **(E)** Quantification of the density of AMPAR-containing intracellular compartments in dendritic segments. Exemplary images of dendrite segments of control neurons and neurons overexpressing TARPγ-8 or CKAMP44a are shown **(left panel)** (scale bar = 2 μm). The mean density of dendritic SEP-GluA1-positive intracellular structures in neurons overexpressing either auxiliary subunit was quantified **(right panel)**. Data are mean ± SEM. *n*-values are given in panel (B) and apply to all data shown. Student’s *t*-test was used for statistical analysis. ***p < 0.001.

To learn about the role of auxiliary subunits in local dendritic turnover of AMPARs, we studied constitutive receptor insertion in dendrites of neurons that co-expressed either TARPγ-8 or CKAMP44 together with SEP-GluA1. We generally used the CKAMP44a splice variant when testing the effect of CKAMP44, as its function has been best characterized in earlier studies (von Engelhardt et al., 2010; Khodosevich et al., 2014). The employed expression vectors for TARPγ-8 and CKAMP44a contained an additional bicistronic open-reading-frame for NLS-tdTomato, which allowed for an easy identification of double-transfected neurons. Note that NLS-tdTomato is targeted to the cell nucleus because of its nuclear localizing sequence and thus does not interfere with imaging experiments at dendritic regions. Our analysis revealed a dramatic reduction in the number of AMPAR insertion events per minute (total recording interval 6 min) in dendritic structures of neurons overexpressing TARPγ-8 or CKAMP44a in comparison to controls (Figures 2A, B). To account for the varying geometry of the imaged dendrites in our experiments, it is necessary to normalize the observed fusion rate to the number of available dendritic REs, from which the vast majority of receptor delivery events originates. SEP-GluA1-positive REs were identified by brief application of NH_4_Cl at the beginning of each experiment. Similar to the absolute fusion rates, the normalized event frequencies were severely reduced by more than 80% in neurons overexpressing either auxiliary subunit (Figure 2C), which confirms that the observed effects are not simply due to an altered distribution or density of dendritic REs. In scatter plots of insertion frequency versus RE number (Figure 2D), we observed a clearly decreased slope of the regression lines for neurons overexpressing TARPγ-8 or CKAMP44a compared to controls, which implies that available dendritic REs exhibit a lower propensity to undergo fusion. That said, we also noted a moderately reduced density of SEP-GluA1-containing intracellular structures in the dendrites of neurons overexpressing auxiliary subunits (Figure 2E), which may point to additional changes in the organization of intracellular AMPAR pools.

**FIGURE 3:**
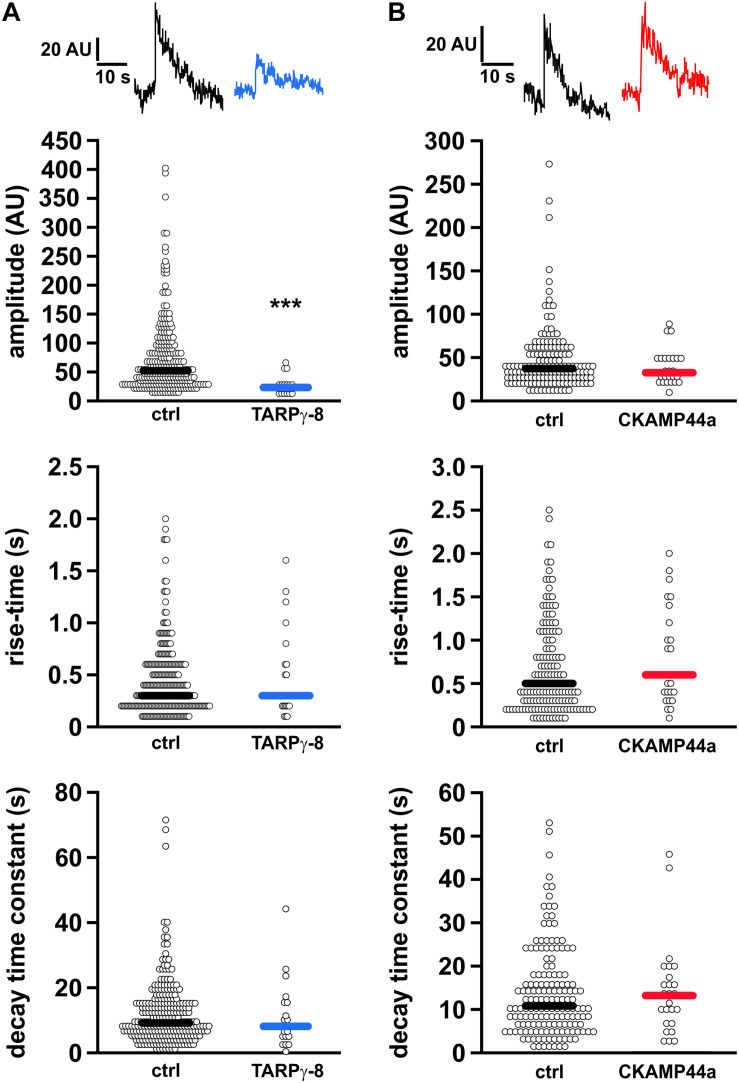
Kinetic analysis of local SEP-fluorescence transients during receptor surface delivery. **(A)** Quantification of amplitude **(upper panel)**, rise time **(middle panel)**, and decay time constant **(bottom panel)** of the SEP-fluorescence transients observed during fusion events in controls (267 events) and neurons overexpressing TARPγ-8 (21 events). Shown are beeswarm plots of the binned data. The median values are indicated by the thick horizontal lines in each plot. Only the fluorescence amplitude of fusion events in neurons containing elevated TARPγ-8 levels was significantly reduced. **(B)** Analogous analysis for fusion events in neurons overexpressing CKAMP44a (25 events) and corresponding controls (156 events). Statistical analysis was performed using Mann–Whitney Rank Sum Test. ***p < 0.001.

In view of the decreased dendritic receptor re-insertion rates, we wondered about potential kinetic alterations of the residual fusion events in neurons overexpressing either auxiliary subunit. Thus, we carefully analyzed amplitude (peak fluorescence), rise-time, and decay kinetics of the individual fusion events. We found that the median amplitude of insertion events was significantly reduced in neurons overexpressing TARPγ-8 in comparison to controls (ctrl: 52.234 AU, *n* = 267; TARPγ-8: 23.184 AU, *n* = 21; *p* < 0.001, Mann–Whitney rank sum test), whereas median values of onset and decay time constant remained unchanged (Figure 3A). As for CKAMP44a overexpression, the signal kinetics showed a very slight, but non-significant decrease in the median of event amplitude (ctrl: 37.243 AU, *n* = 156; CKAMP44a: 32.569 AU, *n* = 25; *p* = 0.468, Figure 3B). Event decay time constant and rise-time were also not significantly different compared to controls.

In sum, our data demonstrates that the dendritic insertion rate of AMPARs is severely reduced upon elevated expression of TARPγ-8 or CKAMP44a, suggesting that these auxiliary subunits could directly or indirectly delay AMPAR re-insertion from REs.

### High Levels of TARPγ-8 or CKAMP44a Reduce the Pool of AMPARs in REs

**FIGURE 4:**
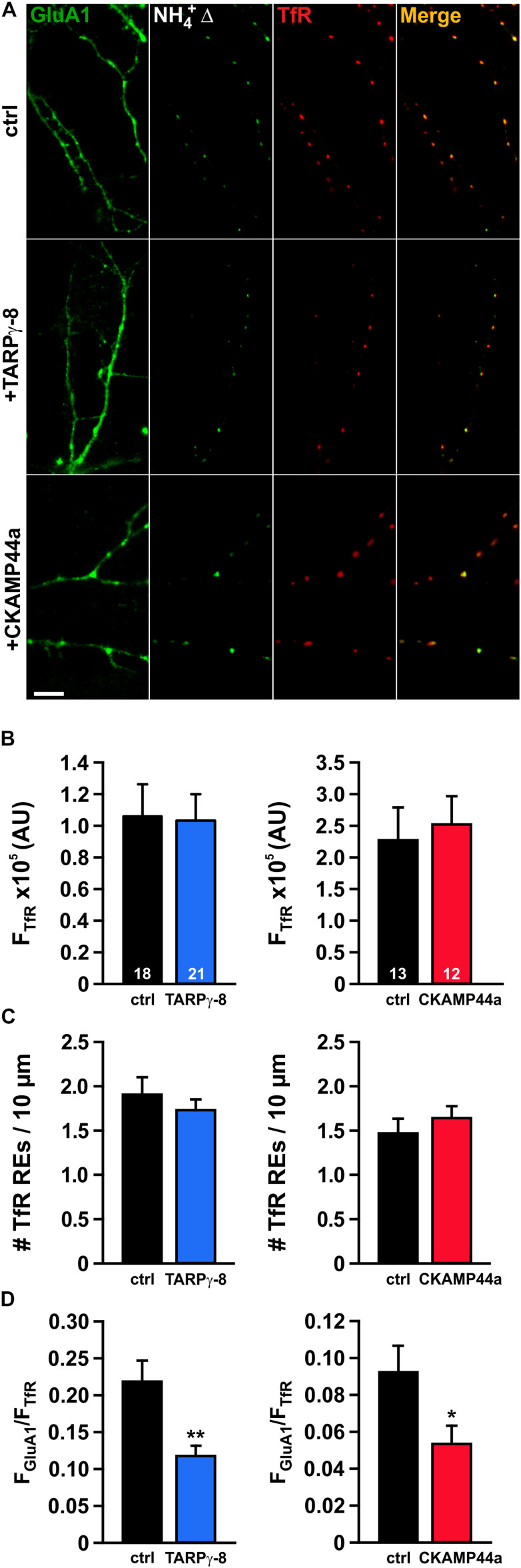
The AMPAR pool in recycling endosomes is decreased in neurons overexpressing auxiliary subunits. **(A)** Exemplary images of dendritic segments of neurons transfected with SEP-GluA1 and TfR-tagRFPt (top row), or neurons additionally expressing either TARPγ-8 (middle row) or CKAMP44a (bottom row) (scale bar = 10 μm). To highlight AMPARs in intracellular dendritic compartments, neurons were treated with NH_4_^+^, and a difference image was calculated by subtraction of baseline fluorescence (NH_4_^+^Δ). **(B)** Averaged median intensity of TfR-tagRFPt puncta is shown for neurons overexpressing TARPγ-8 **(left panel)** and neurons overexpressing CKAMP44a **(right panel)**. **(C)** Quantification of the density of TfR-positive puncta per 10 μm dendrite in neurons overexpressing either auxiliary subunit. **(D)** The intensity ratio of the SEP-GluA1 signal to the TfR-tagRFPt signal was calculated for each detected RE-puncta and subsequently averaged for each neuron. The mean ratios for each experimental group are shown. Depicted errors are SEM. *n*-values shown in panel (B) also apply to the other bar graphs. Statistical analysis was assessed with Student’s *t*-test. *p < 0.05;**p < 0.01.

As the endosomal lumen should be vastly neutralized during transient fusion pore opening, the observed reduction of the event amplitude in neurons overexpressing TARPγ-8 points to a decreased population of SEP-GluA1-containing AMPARs within dendritic REs. To investigate the status of the endosomal AMPAR pool in neurons overexpressing either type of auxiliary subunit, we again labeled REs using TfR-tagRFPt and visualized the GluA1-containing RE subset by brief application of NH_4_^+^ (Figure 4A). Unsurprisingly, the general organization of TfR-tagRFPt-marked REs in dendrites appeared unchanged by overexpression of auxiliary subunits, as reflected by a similar mean fluorescence intensity of TfR-tagRFPt in REs under all experimental conditions (Figure 4B). Moreover, our analysis of the number of TfR-tagRFPt-marked REs per μm dendrite did not reveal any changes induced by overexpression of either type of auxiliary subunit (Figure 4C). In order to estimate the AMPAR content in dendritic REs, we superimposed the intracellular SEP-GluA1 signal (“NH_4_^+^Δ”-image) onto the tagRFPt-fluorescence image and calculated the fluorescence ratio of the SEP signal and tagRFPt signal for each individual RE. The mean ratio between SEP and tagRFPt fluorescence (F_GluA__1_/F_TfR_) was significantly smaller for REs in neurons overexpressing TARPγ-8 than in controls (Figure 4D), indicating that TARPγ-8 can decrease the AMPAR pool in REs. When the same analysis was performed for REs in dendrites of CKAMP44a-overexpressing neurons, we also observed a significant, albeit less pronounced reduction in the mean F_GluA__1_/F_TfR_ ratio. Thus, our data suggest that overexpression of either auxiliary subunit steers receptor trafficking away from dendritic REs, which may explain the reduced fusion event amplitude in TARPγ-8-expressing cells and may at least partly account for the lower event frequency due to an increased number of low intensity events that evade detection.

### Overexpression of Auxiliary Subunits Decelerates Internalization of Extrasynaptic AMPARs

The observed attenuation of the AMPAR pool in dendritic REs strongly suggests that TARPγ-8 or CKAMP44a are involved in the regulation of local AMPAR cycling. On a mechanistic level, association with auxiliary subunits could either reduce their internalization rate or alternatively facilitate their sorting to late endosomes/lysosomes, thereby designating more receptors for degradation. To directly address the first possibility, we investigated the basal internalization rate of AMPAR in live-cell imaging experiments using a “pulse-chase”-like imaging strategy. For this purpose, we constructed a new reporter, in which a self-labelling HaloTag (Promega) was fused to the N-terminus of GluA1 (Supplementary Figure 1A). As a modified hydrolase, the HaloTag can catalyze covalent binding of a chloroalkane group to its enzymatic domain, enabling versatile staining of tagged receptors with engineered fluorescent HaloTag substrates (Los et al., 2008). As the N-terminally localized HaloTag-domain of surface AMPARs is freely accessible to substrates in the extracellular medium, we incubated neurons with a membrane-impermeable fluorescent HaloTag-ligand (Alexa Fluor 488-Ligand, G1001) to selectively label the surface receptor pool in HaloTag-GluA1-transfected neurons. Preparatory experiments established that a reduced ambient temperature (15°C) during the staining interval (35–40 min) sufficiently decelerated endocytosis to prevent premature receptor internalization. Repeated imaging of stained neurons (incubated at 37°C) with confocal microscopy allowed us to characterize AMPAR internalization based on the progressive fluorescence decline at the plasma membrane. To quantify the decay of HaloTag-GluA1 fluorescence on the surface, we traced plasma membrane segments of dendrites with line scans. The image regions surrounding the central line scan were transformed into a rectangular image (ImageJ; Kocsis et al., 1991), whose pixel lines represent orthogonal fluorescence profiles across the plasma membrane (Supplementary Figure 1B). For each time point, all profiles of the transformed image section were averaged and the resulting peak membrane fluorescence was quantified. To yield reliable data on the fluorescence decay, we analyzed multiple dendritic segments in each neuron and calculated the mean relative fluorescence intensity. The general receptor internalization kinetics observed by this method is shown in Supplementary Figure 1B (right panel).

**FIGURE 5:**
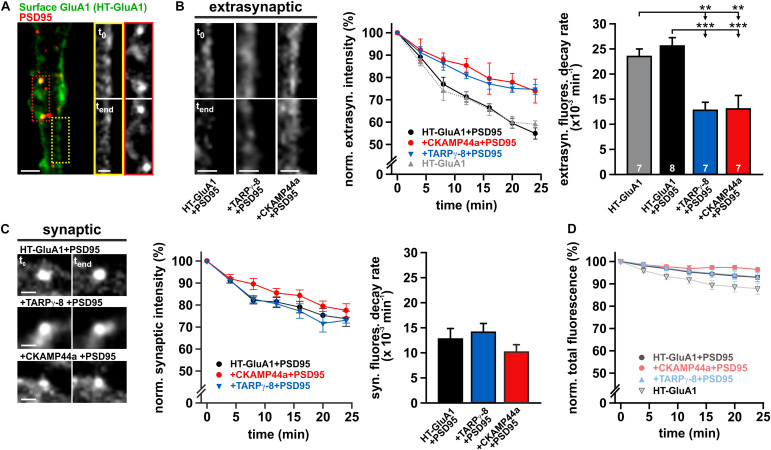
Overexpression of auxiliary subunits reduces basal AMPAR internalization. **(A)** Confocal image of a representative dendritic segment from a neuron that was co-transfected with HaloTag (HT)-GluA1 and PSD95-tagRFPt to separately follow extrasynaptic and synaptic receptor turnover (scale bar = 4 μm). Cropped detail pictures show labeled GluA1-receptors at the marked extrasynaptic (yellow box) and synaptic regions (red box) directly after staining (t_0_) and after 24 min incubation (t_end_) (scale bar = 2 μm). **(B)** Example images show the internalization of extrasynaptic receptors at dendritic segments of control neurons and cells overexpressing TARPγ-8 or CKAMP44a (scale bars = 2 μm). The internalization kinetics of labeled surface AMPARs at extrasynaptic sites were quantified for each experimental group **(middle panel)**. The averaged fluorescence decay profile for controls (+PSD95) is depicted in black, for neurons overexpressing TARPγ-8 in blue, and for cells overexpressing CKAMP44a in red. The gray, dotted line outlines the fluorescence decay in control cells not expressing the synaptic marker PSD95-tagRFPt. Data was fit by monoexponential functions to determine the internalization rate **(right panel)**. **(C)** Analogous analysis of fluorescence decay for labeled HT-GluA1-containing AMPARs at synaptic sites (scale bars = 2 μm). **(D)** Bleaching was estimated by quantifying the relative loss of summed fluorescence of the full image. All depicted data are mean ± SEM. *n*-values for the experimental groups shown in panel (B) also apply to all other panels. Statistical analysis was performed with one-way ANOVA and Tukey *post hoc* test. **p < 0.01; ***p < 0.001.

Taking into account that auxiliary subunits are known to support synaptic aggregation of AMPARs (Buonarati et al., 2019), overexpression of TARPγ-8 or CKAMP44a could simply affect receptor turnover by expanding and stabilizing the pool of synaptic AMPARs. Therefore, we employed a fluorescently labeled variant of the synaptic scaffolding protein PSD95 (PSD95-tagRFPt) to mark postsynaptic sites and separately quantified the internalization rate of extrasynaptic and synaptic receptors. The fluorescence intensity of synaptic HaloTag-labeled AMPARs was only measured at sites outlined by PSD95-tagRFPt fluorescence, while the extrasynaptic HaloTag-GluA1-signal was analyzed by tracing membrane fluorescence with longitudinal line scans (≥3 μm) in membrane sections that were free of PSD95-tagRFPt puncta (Figure 5A). Interestingly, overexpression of TARPγ-8 or CKAMP44a differentially affected the internalization of HaloTag-labeled surface receptors at synaptic and extrasynaptic sites. The fluorescence decline over time at extrasynaptic sites was significantly faster in controls than in experiments with neurons overexpressing either auxiliary subunit (Figure 5B). Decay kinetics of extrasynaptic surface fluorescence were approximated by monoexponential functions and showed significantly increased time constants for internalization (i.e., decreased uptake rates) in the presence of auxiliary subunits compared to controls (Figure 5B, right panel). Photobleaching over the recording interval was estimated by total fluorescence loss in the field of view, following the rationale that endocytosis of labeled surface AMPARs should only re-distribute but not eliminate the fluorescence signal. The overall fluorescence loss that could be attributed to bleaching was comparably low, confirming that receptor internalization is indeed the primary reason for the observed fluorescence decline at the plasma membrane (Figure 5D).

The uptake of synaptic AMPARs was noticably slower than the internalization of extrasynaptic AMPARs, as indicated by the reduced internalization rates of synaptic receptors reported by monoexponential fits (Figure 5C). Moreover, no alterations in fluorescence decay kinetics were detectable in neurons overexpressing TARPγ-8 or CKAMP44a. Monoexponential fits yielded receptor internalization rates that were indistinguishable for all tested conditions. Although the internalization rate of synaptic AMPARs is putatively limited by the dissociation of receptors from scaffolding proteins and their subsequent escape from the PSD (Bats et al., 2007; Nair et al., 2013), our data demonstrates that increasing the amount of either auxiliary subunit in AMPAR complexes did not affect the dynamics of synaptic receptors.

In sum, our data on receptor turnover kinetics suggests that an increased association of the tested auxiliary subunits with AMPAR core complexes reduces the internalization rate of extrasynaptic receptors. Strikingly, both, TARPγ-8 and CKAMP44a, seem equally effective in delaying receptor uptake, which suggests a related mechanism in spite of the striking structural differences of both types of subunits.

### Surface Pool and Dendritic Distribution of AMPARs Are Differentially Affected by Overexpression of TARPγ-8 or CKAMP44a

**FIGURE 6:**
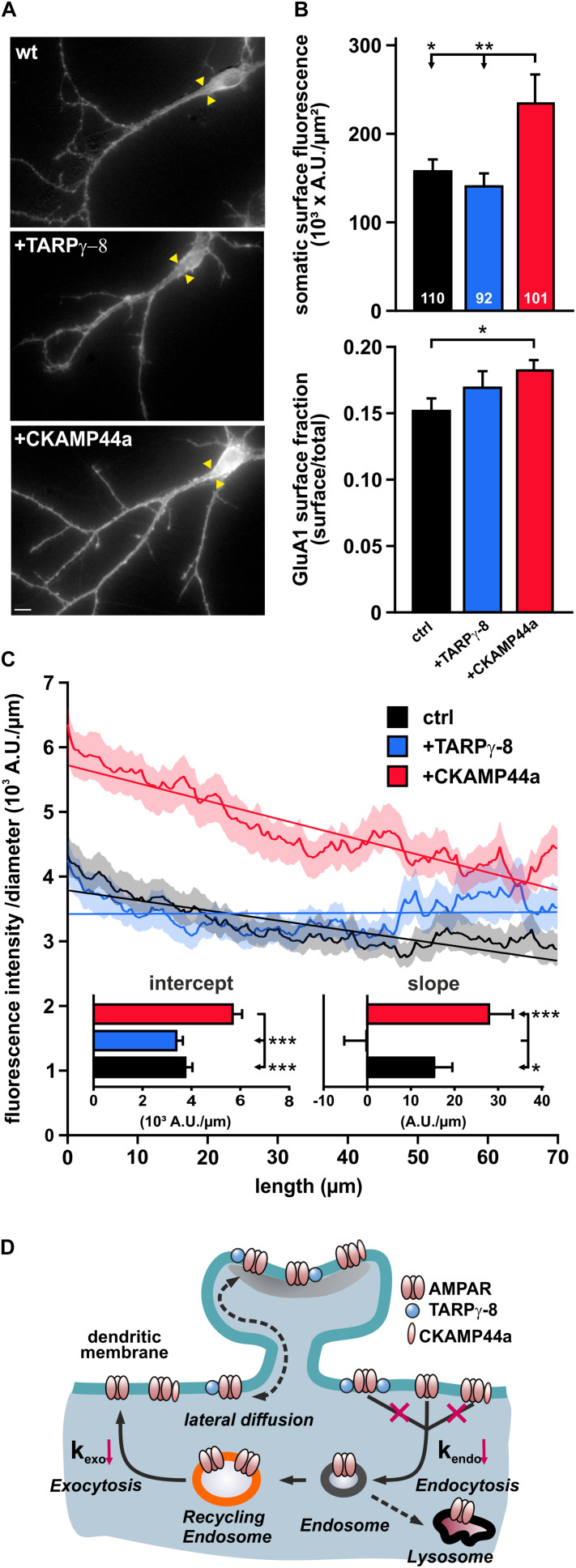
Overexpression of auxiliary subunits differently affects AMPARs surface distribution. **(A)** Exemplary difference images show the “isolated” surface SEP-GluA1 signal in control neurons and cells overexpressing either TARPγ-8 or CKAMP44a (scale bar = 10 μm). Yellow arrowheads mark the assumed origin of the neurite, which was used as the starting point for the analysis of receptor distribution along the main dendrite branch. **(B)** Mean fluorescence density in somatic areas was quantified for neurons with high abundance of TARPγ-8, CKAMP44a, or controls **(upper panel)**. To investigate the distribution of AMPARs between intracellular compartments and surface, the mean fluorescence ratio F_surface_/F_total_ was calculated for the somatic region **(lower panel)**. **(C)** The specific distribution profile of SEP-GluA1-fluorescence along dendrites was investigated by integrating individual pixel columns of a transformed image of each dendritic branch, wherein the x-axis was aligned to the longitudinal axis of the dendrite. Fluorescence at each position was normalized to the diameter of the dendrite and plotted against the distance from the soma. Shown are averaged distribution profiles for controls (black), TARPγ-8 (blue), and CKAMP44a-overexpressing neurons (red). SEM values are indicated by the areas of lighter color surrounding each curve. Curves were fit by linear functions, and resulting fit parameters are shown in insets. **(D)** The cartoon summarizes the effects of overexpression of auxiliary subunits on the dendritic turnover of AMPARs. Our data suggest that the basal AMPAR internalization rate (k_endo_) and the corresponding reinsertion rate (k_exo_) are decreased in response to overexpression of TARPγ-8 or CKAMP44a. All depicted data are mean ± SEM. n-values are given in panel (B) and apply to all data. Statistical analysis was performed with ANOVA and Tukey’s multiple comparisons test. ***p < 0.001, **p < 0.01, *p < 0.05.

The reduced rates of AMPAR internalization and re-insertion in neurons overexpressing TARPγ-8 or CKAMP44a might alter the steady-state density and distribution of surface receptors. To investigate potential effects of these auxiliary subunits on the AMPAR surface pool, we quenched the fluorescence of SEP-GluA1-containing receptors on the plasma membrane by application of acidic extracellular solution (pH 5.5) and calculated difference images (I_surface_ = I_before_ – I_pH__5_._5_) to visualize all surface receptors (Figure 6A). We first quantified the receptor density at the soma and found that overexpression of CKAMP44a—but not TARPγ-8—resulted in a significant increase in SEP-GluA1-fluorescence per μm^2^ plasma membrane compared to controls (Figure 6B). We also normalized the surface receptor pool to the overall expression of SEP-GluA1-containing AMPARs in the respective neurons, calculating the surface fraction F_surface_/F_total_ after unquenching SEP-GluA1-containing receptors in acidic intracellular compartments by application of a NH_4_^+^-containing solution. Noteworthy, we found a significant increase in the fraction of surface receptors in neurons overexpressing CKAMP44a (Figure 6B), which suggests that the increased surface pool is caused by a redistribution of receptors and is not simply due to a rise in total expression (ctrl: 1216460 ± 85917 AU/μm^2^; TARPγ-8: 940704 ± 77469 AU/μm^2^; CKAMP44a: 1264599 ± 93949 AU/μm^2^).

To investigate receptor distribution along dendrites, we selected proximal segments of dendrites with mild curvature and a minimal length of 70 μm for further analysis. The main dendrite branch and surrounding image regions (±8.6 μm) were transformed into a rectangular image (ImageJ; Kocsis et al., 1991), in which the “straightened” dendrite was perfectly aligned with the x-axis of the image. SEP fluorescence in each individual pixel row of the image was integrated to correlate dendrite length with the corresponding receptor expression. In order to account for the progressively decreasing surface of the cylindrical dendrite, we also normalized fluorescence to dendrite diameter. As plots of the normalized surface fluorescence against the distance from soma showed a shallow fluorescence decline with dendrite length (Figure 6C), we fit each individual fluorescence profile with a simple linear function. The averaged y-intercept of the fit lines corresponded to the somatic AMPAR surface density and was significantly increased in neurons overexpressing CKAMP44a, replicating our earlier findings for the somatic region. Moreover, the fluorescence decline along the dendrites (line slope) of CKAMP44a-overexpressing neurons tended to be steeper than in controls. Neurons containing a higher abundance of TARPγ-8 appeared undistinguishable from controls at first glance but showed almost no fluorescence decline along the dendrite, as indicated by a very shallow slope of the fit line (Figure 6C). This suggests that overexpression of TARPγ-8 induced a subtle but significant shift in AMPAR distribution to more distal positions.

In sum, these results demonstrate that the two tested auxiliary subunits, CKAMP44a and TARPγ-8, alter the surface receptor pool in very different ways despite exerting similar effects on dendritic AMPAR turnover. While the reduced turnover of surface AMPARs in neurons overexpressing CKAMP44a putatively contributes to an overall elevation in surface receptor expression, TARPγ-8 overexpression rather subtly strengthens the expression of tagged AMPARs in dendrites.

## Discussion

In different regions of the central nervous system, AMPAR channels are associated with specific sets of accessory proteins and auxiliary subunits that regulate channel gating and receptor trafficking (Schwenk et al., 2012, 2014; Jacobi and von Engelhardt, 2017). While previous work clearly demonstrated that auxiliary subunits promote ER export and receptor maturation (Tomita et al., 2003; Greger et al., 2007; Harmel et al., 2012), potential functions of auxiliary subunits in local trafficking and dendritic turnover of AMPAR complexes have gained little attention. In this study, we present evidence for a critical involvement of the auxiliary subunits TARPγ-8 and CKAMP44a in controlling constitutive endosomal cycling of AMPARs in the dendrites of hippocampal neurons. We show that an increased abundance of either of these auxiliary subunits results in a reduced turnover of AMPARs, prolonging the surface lifetime of receptors by delaying receptor uptake (Figure 6D).

In mammals, TARPγ-8 and CKAMP44a are both expressed within hippocampal neurons: CKAMP44a is mainly restricted to dentate gyrus (DG) granule cells, while TARPγ-8 expression is spread broadly across pyramidal cells (CA1–CA3) and DG granule cells (Fukaya et al., 2006; von Engelhardt et al., 2010). Here, we pursued the experimental approach to alter the composition of receptor complexes by overexpression of TARPγ-8 or CKAMP44a and to analyze resulting effects on dendritic AMPAR trafficking. As TARPs do not occupy all four binding slots in naïve receptor complexes (Shi et al., 2009; Kim et al., 2010), overexpression of TARPγ-8 should cause a shift toward assemblies with higher TARPγ-8 contribution. In line with this idea, earlier work demonstrated that overexpression of TARPγ-8 in hippocampal neurons would significantly increase extrasynaptic AMPAR surface density (Rouach et al., 2005), suggesting that more AMPAR complexes included TARPγ-8 and thus underwent forward trafficking. The restricted expression pattern and relatively low abundance of CKAMP44a in the hippocampus also prompt the expectation that overexpression of this particular subunit should generally result in an increased fraction of CKAMP44a-containing receptors in most neurons. Overexpression of CKAMP44a in DG granule cells has indeed been shown to increase the density and gating properties of extrasynaptic AMPARs as well as mEPSC amplitudes (Khodosevich et al., 2014), suggesting that more receptor complexes incorporated CKAMP44a. To assay AMPAR trafficking in live-cell imaging experiments we co-expressed differently tagged variants of GluA1 with TARPγ-8 or CKAMP44a using independent expression vectors. As tetrameric AMPAR channels supposedly associate with up to four auxiliary subunits (as suggested for TARPs), we transfected neurons with a balanced ratio of expression plasmids, allowing for matched expression levels and thus receptor complexes containing multiple copies of either expressed auxiliary subunit. While overexpression of CKAMP44a increased the surface expression of GluA1-containing receptors in our experiments in accord with previous work (Khodosevich et al., 2014), overexpression of TARPγ-8 did not affect forward trafficking to the surface under our conditions in contrast to previous findings (Rouach et al., 2005). Most likely, this is due to differences in the experimental settings, as Rouach et al. (2005) employed a Semliki Forest virus-based expression system, which boosts protein levels within mere hours after infection, producing a large excess of TARPγ-8.

In analogy to earlier experimental strategies (Yudowski et al., 2007; Lin et al., 2009), we visualized AMPAR trafficking by expression of a GluA1 subunit with an N-terminal SEP-tag, whose pH-induced fluorescence changes report milieu transitions occurring during organelle fusion. TARPγ-8 and CKAMP44a were shown to efficiently bind to and modulate GluA1-homomeric as well as GluA1/GluA2-heteromeric channels (Cho et al., 2007; Khodosevich et al., 2014; Herguedas et al., 2019; von Engelhardt, 2019), assuring that potential trafficking effects of auxiliary subunits can be monitored by our GluA1-based reporters. Noteworthy, SEP-GluA1 overexpression will to some extent produce calcium-permeable GluA1-homomers, which are believed to follow specific activity-dependent trafficking routes in the context of synaptic plasticity (for review see e.g., Hanley, 2014; Henley and Wilkinson, 2016). That said, even GluA1-homomers undergo normal turnover similar to receptors of other subunit composition despite the absence of crucial regulatory elements for clathrin-mediated endocytosis that are exclusively found in the GluA2 C-terminus (Biou et al., 2008; Panicker et al., 2008).

Our imaging data obtained with SEP-GluA1 show a dramatic decline in spontaneous AMPAR insertion rate in neurons with high abundance of TARPγ-8 or CKAMP44a, which suggests a regulatory role of auxiliary subunits in basal dendritic AMPAR cycling. The majority of insertion events in our experiments lasted several seconds and thus was reminiscent of so-called “display events” or “persistent events,” as described in several earlier studies (Yudowski et al., 2007; Jullie et al., 2014; Roman-Vendrell et al., 2014). These long-lasting events are thought to comprise two phases, an initial stage characterized by a fluorescence decay due to dispersion of newly inserted receptors into the plane of the plasma membrane and a subsequent post-closure stage, wherein the fluorescence decay is primarily caused by re-acidification of the resealed organelle (Jullie et al., 2014). In accordance with this idea, we found that TfR-tagRFPt-signals remained remarkably stable during fusion events, indicating that fusion pore opening generally does not lead to RE collapse into the plasma membrane. Intriguingly, we did not observe kinetic alterations of the SEP fluorescence waveform in neurons overexpressing auxiliary subunits, which indicates that fusion mode and fusion pore behavior were largely unchanged. Thus, the apparent drop in spontaneous event frequency is rather explained by a decreased pool of AMPARs in REs, rendering a substantial number of RE fusion events undetectable, and/or an inhibitory regulatory effect on the fusion propensity of REs. Although the fraction of subthreshold events is hard to estimate, the observed 40% reduction in median SEP-GluA1 fluorescence in REs seems substantial enough to severely affect detection at the lower end of the intensity distribution. Note that a reduced event amplitude was not observed in neurons overexpressing CKAMP44a, albeit the AMPAR pool in REs was almost halved, which might be explained by an elevated detection threshold due to the increased surface fluorescence in these cells. Normalizing fusion frequency to the number of detectable REs in the corresponding field of view only slightly lessened the sharp drop in fusion rate observed in neurons overexpressing the auxiliary subunits, which argues strongly in favor of an additional negative regulation of RE fusion probability by the auxiliary subunits. It might be speculated that the cytosolic domains of the auxiliary subunits could mediate such a regulative function, as association of AMPAR subunits with scaffolding proteins like GRIP or SAP97 has previously been proposed to establish binding platforms for recruitment of motor proteins or signaling complexes that facilitate cycling back to the plasma membrane (Parkinson and Hanley, 2018).

Our experiments with a new GluA1 variant carrying an extracellular self-labeling HaloTag demonstrated that overexpression of TARPγ-8 or CKAMP44a reduced constitutive local receptor turnover in dendrites, readily explaining the decreased AMPAR population in endosomal compartments. The dehalogenase domain of HaloTag mediates covalent binding of synthetic fluorophores allowing for a selective staining of surface AMPARs by transient application of a membrane-impermeable ligand in “pulse-chase”-paradigms. The constitutive AMPAR turnover measured within 20–30 min using HaloTag-GluA1 at extrasynaptic sites was largely comparable to results reported for GluA1-internalization based on immunocytochemical assays (Lee et al., 2004). Note that most previous studies did not distinguish between extrasynaptic and synaptic receptor pools, possibly delivering lower estimates for receptor internalization rate due to the inclusion of the more stable synaptic receptor population (Ehlers, 2000). Our live-cell imaging experiments with HaloTag-GluA1 showed that the constitutive uptake of labeled receptors from extrasynaptic sites was significantly delayed in neurons overexpressing TARPγ-8 or CKAMP44a, whereas fluorescence decay within synaptic areas was unaltered in comparison to controls. Noteworthy, CKAMP44a and TARPγ-8 have both been implicated in synaptic anchorage of AMPARs via their PDZ-binding motifs (Sumioka et al., 2011; Khodosevich et al., 2014), and thus complexes with a higher copy number of these auxiliary subunits should engage in stronger scaffold interactions at synaptic sites. This might trap AMPAR in synaptic nanodomains (Bats et al., 2007; Nair et al., 2013) and delay their internalization at peripheral endocytic zones (Lu et al., 2007). Recent findings have also emphasized the role of TARP phosphorylation in synaptic AMPAR trafficking, as phosphorylated C-terminal motifs have been proposed to positively regulate TARP binding to synaptic scaffold proteins (Sumioka et al., 2010), while dephoshorylated TARPs associate with the μ2 subunit of AP-2 and promote AMPAR recruitment to clathrin-coated pits (Matsuda et al., 2013). A higher number of C-terminal domains of TARPs in complexes due to TARPγ-8 overexpression should therefore change the propensity of receptors to remain attached to synaptic scaffolding proteins, as more interactions must be broken to escape synaptic clusters. Dispelling these mechanistic ideas, we could, however, not detect any changes in synaptic AMPAR dynamics, arguing that the phosphorylation state and/or the specific composition of synaptic AMPAR complexes prohibits the expected interactions and increased stability of synaptic AMPARs under our experimental conditions.

While clathrin-mediated endocytosis is thought to be the central pathway for activity-dependent removal of synaptic AMPARs during LTD (Hanley, 2018; Moretto and Passafaro, 2018), recent evidence suggests that constitutive receptor internalization is generally mediated by a clathrin- and dynamin-independent mechanism (Glebov et al., 2015; Zheng et al., 2015). Given the scarce information on clathrin-independent AMPAR internalization, a specific role of auxiliary subunits in this process is currently hard to define. Since CKAMP44a and TARPγ-8 are structurally diverse but exert similar effects on AMPAR trafficking, one might speculate that the presence of either subunit in complexes should affect receptor internalization via a rather general mechanism, possibly by limiting access to essential accessory proteins or specific membrane segments dedicated to constitutive endocytosis. In this respect, it is highly interesting that the auxiliary subunit GSG1L reduces surface expression by facilitating AMPAR endocytosis (Gu et al., 2016). As structural data on AMPAR/GSG1L assemblies indicates that GSG1L is occupying an overlapping binding slot with TARPs (Twomey et al., 2017), strong overexpression of TARPγ-8 could competitively reduce GSG1L incorporation in complexes and could thereby stall GSG1L-induced receptor internalization. Inconsistently, however, GSG1L is rarely found in hippocampal AMPAR complexes (Schwenk et al., 2014) and thus should be only of minor importance for constitutive endocytosis in our model system. Moreover, GSG1L has been classified as a subunit of the inner receptor complex, while CKAMP44a is believed to occupy a different slot as an outer core protein (Schwenk et al., 2012), which strongly argues against a prominent binding competition. Therefore, a displacement of GSG1L cannot conclusively explain the stabilizing effect of both auxiliary subunits on extrasynaptic surface AMPARs.

Providing another perspective on the role of auxiliary subunits in AMPAR internalization, Tomita et al. (2004) reported in an early study that the TARP isoform γ-3 dissociates from receptor complexes upon agonist-induced endocytosis and is subsequently recycled with a slower time course. These findings potentially imply that AMPARs need to shed off auxiliary subunits and/or other complex components before undergoing internalization. If this were true, such requirement would readily explain why increasing the abundance of auxiliary subunits prolongs AMPAR surface lifetime, as an excess of auxiliary subunits should strengthen complex integrity due to the law of mass action. Dissociation of TARPs from complexes has previously been postulated to occur during receptor desensitization and to lead to internalization (Morimoto-Tomita et al., 2009). On a related note, it was also demonstrated that cross-linking AMPARs with TARPγ-2 precluded a glutamate-induced increase in lateral receptor diffusion rate (Constals et al., 2015), indicating a regulation of receptor surface mobility via dynamic association of auxiliary subunits. Therefore, it stands to reason that decreased receptor surface mobility in the presence of auxiliary subunits could limit its effective incorporation in protein complexes mediating constitutive endocytosis.

Taken together, we found that increased numbers of CKAMP44a and TARPγ-8 subunits in receptor complexes prolong the surface lifetime of extrasynaptic receptors by delaying constitutive internalization. To some extent, the loss of this stabilizing function might also be responsible for the dramatic reduction of the extrasynaptic receptor density in hippocampal neurons of TARPγ-8^–/–^ animals (Rouach et al., 2005), as an accelerated receptor uptake might aggravate the effect of defective forward trafficking on the surface receptor pool in knock-out neurons. Similarly, an increased AMPAR internalization rate might contribute to the moderate reduction in the surface receptor pool of dentate gyrus neurons in CKAMP44^–/–^ animals (Khodosevich et al., 2014). Given the fundamental role of dynamic receptor cycling in synaptic plasticity, it will be highly interesting to investigate in future experiments which specific motifs and interactions of auxiliary subunits control dendritic receptor internalization.

## Data Availability Statement

The original contributions presented in the study are included in the article/Supplementary Material, further inquiries can be directed to the corresponding author.

## Author Contributions

RM, DB, and UB designed the research. AH, NV, and AS performed the research. AH, NV, and RM analyzed the data. RM wrote the manuscript with input of UB, DB, NV, and AH. All authors contributed to the article and approved the submitted version.

## Conflict of Interest

The authors declare that the research was conducted in the absence of any commercial or financial relationships that could be construed as a potential conflict of interest.

## Publisher’s Note

All claims expressed in this article are solely those of the authors and do not necessarily represent those of their affiliated organizations, or those of the publisher, the editors and the reviewers. Any product that may be evaluated in this article, or claim that may be made by its manufacturer, is not guaranteed or endorsed by the publisher.
