# Supplementary material for: Auxiliary Subunits Regulate the Dendritic Turnover of AMPA Receptors in Mouse Hippocampal Neurons
Source: Front Mol Neurosci. 2021 Aug 23;14:728498. doi: 10.3389/fnmol.2021.728498 (PMC8419334; doi:10.3389/fnmol.2021.728498)
Supplement: Supplementary file 1 [file Image_1.pdf]

Supplementary Material

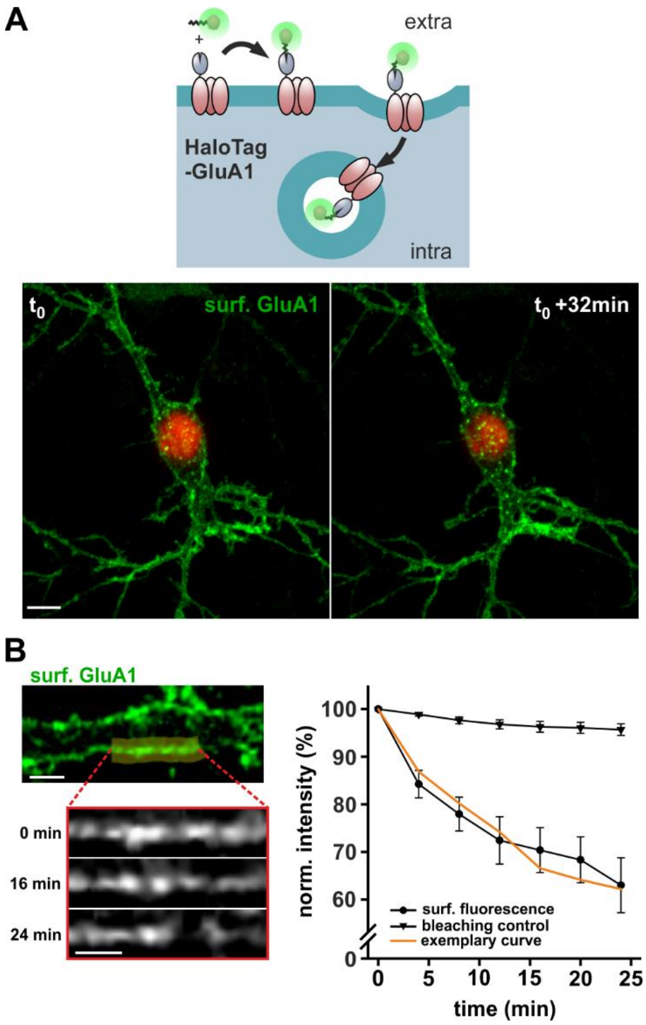

Supplementary Figure 1. Analysis of AMPAR uptake using HaloTag-GluA1 as a reporter. **(A)** The cartoon illustrates the structure of HaloTag-GluA1, which carries a self-labelling protein fusion tag at the extracellular N-terminus and allows for selective staining of surface AMPARs by application of a membrane-impermeable fluorescent ligand. Example images show a neuron directly after staining (t_0) and after 32 min rest (scale bar = 20 µm). Note the increased number of stained, punctate-shaped intracellular organelles after prolonged incubation, indicating the ongoing internalization of labelled surface AMPARs. **(B)** Quantification of receptor internalization: Appropriate membrane sections in confocal images were manually traced with line scans (scale bar = 5 µm), and surrounding regions were transformed to obtain “straigthened” images of the membrane section. Detail pictures show images of the same membrane region at three different consecutive time points (scale bar = 3 µm). Peak fluorescence values along the membrane in each image were averaged as a measure for receptor surface density. The plot shows the observed surface fluorescence decline in the example region (orange line) as well as the averaged decay curve from a dataset of 7 neurons. Bleaching was estimated by the decrease of total summed fluorescence in full images. Depicted data are mean ± SEM.
